# The Beneficial Role of Intra Cytoplasmic Morphologically Selected Sperm Injection (IMSI) in Assisted Reproduction

**Published:** 2020

**Authors:** Esmat Mangoli, Mohammad Ali Khalili

**Affiliations:** - Research and Clinical Center for Infertility, Reproductive Science Institute, Shahid Sadoughi University of Medical Sciences, Yazd, Iran

**Keywords:** ICSI, IMSI, Male infertility, Sperm morphology

## Abstract

Since the introduction of intracytoplasmic sperm injection (ICSI), the importance of sperm morphology assessment has been given attention in the assisted reproduction field. It is important to select a good-quality motile spermatozoon for giving a better embryo quality in assisted reproduction technique (ART). In ICSI, sperm morphology evaluation is limited due to its low magnification. However, by using intracytoplasmic morphologically selected sperm injection (IMSI), the selection is done at high magnification of ×6600 using motile sperm organelle morphology examination (MSOME). Therefore, it becomes possible to select a good quality spermatozoon with an intact nucleus that may enhance the pregnancy outcomes. Although all patients can benefit from IMSI, it is important to standardize which techniques (IMSI or ICSI) could be used or which group of patients benefit from IMSI to maximize the efficiency of this advanced technology.

## Introduction

Intracytoplasmic sperm injection (ICSI) was introduced in 1992 for the treatment of male factor infertility. Whenever possible, ICSI is done using morphologically normal spermatozoa selected with the inverted microscope, magnification of ×400 (1). There was linear correlation between the quality of spermatozoa and embryo development and pregnancy outcome. Ideally, only spermatozoa with a higher reproductive capacity are used for ART. These spermatozoa would be viable and mature, structurally complete with high DNA integrity (2, 3). Correlations between reproductive outcomes and sperm morphology and/or other semen characteristics, like motility, concentration, membrane stability, mitochondrial action or DNA fragmentation have been debatable topics in reproductive studies. Among the sperm characteristics, sperm morphology has usually played a key role in determining fertility potential (4, 5).

For the first time, Bartoov et al. introduced the motile sperm organelle morphology examination (MSOME) technique. They assessed nuclear morphology of motile spermatozoa at high magnification in real time (6). For this purpose, they needed a reverse light microscope equipped with high-power differential interference contrast (DIC) optics after an optical magnification of ×1500. Further enrichment by digital imaging permitted attaining a total magnification of up to ×6600. This magnification allows identifying a spermatozoon with a normal nucleus, defined by an oval shape with a smooth configuration and a normal nuclear content and without vacuoles or with vacuoles occupying fewer than 4% of the nucleus (7). Initially, MSOME assessed six sperm structures like acrosome, post-acrosome lamina, nucleus, neck, tail and mitochondria. However, the sperm nucleus seemed to be the significant influencing factor in the ICSI outcome (8). Several publications reported higher pregnancy rates in couples with repeated ICSI failures following the use of spermatozoa with normal nucleus selected at high magnification (9–12).

**Figure 1.**
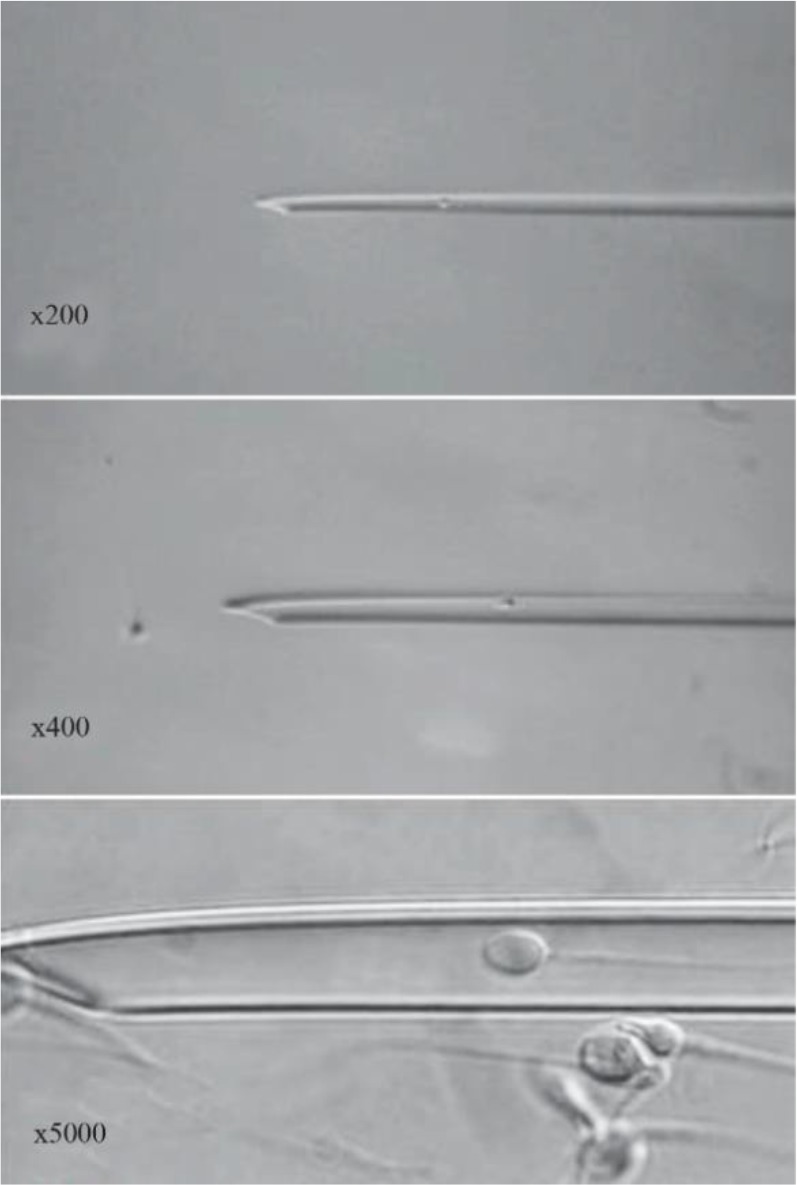
Human spermatozoon: morphological appearance in microinjection pipette, ×200, ×400 and ×6000. The morphological integrity of sperm is clearly visible at ×6000

In addition to the normalization of the head in terms of the shape and size, the presence of the nuclear vacuoles in the sperm head plays an important role in the outcomes, which reduces pregnancy and increases the abortion rate (13, 14). It would be clinically appropriate to describe the frequency of vacuoles within semen samples of a given ICSI population and to know their specific impact on oocyte fertilization, embryo development and implantation. Efforts have been made to detect the origin and structure of these vacuoles in the sperm head. The existence of large vacuoles in the sperm head has been attributed to acrosome status, chromatin condensation, DNA fragmentation and sperm aneuploidy (15). The combination of MSOME technique with a micromanipulation system has allowed the introduction of a modified ICSI procedure, called intracytoplasmic morphologically selected sperm injection (IMSI). This non-invasive system is able to choose the best available motile spermatozoa using the accurate morphological evaluation at high magnification, ranging from ×6600 to ×13,000 with Nomarski optics (Figure 1) (4, 6, 9, 16–19).

On the other hand, the IMSI procedure is very time consuming depending on the quality of the spermatozoa and the number of oocytes to be injected. The cut-off for the acceptable shape of the individual spermatozoa to be selected or deselected by this procedure remains unclear. A number of studies have reported that IMSI is definitely related to implantation and pregnancy rates (9, 20). However, the exact indications for IMSI are still debated. So, in this review, an attempt was made to assess which microinjection technique produces better clinical outcomes in ART.

### IMSI in male factor patients:

IMSI has a central role in collection of information on male infertility. Now, few randomized controlled trials evaluated the benefits of IMSI over the ICSI technique. Some studies showed IMSI procedure is an appreciated choice for the cases with severe teratozoospermia (T) at their first or second attempts. IMSI caused a significantly higher clinical pregnancy rate in these patients (12, 21, 22). Balaban et al. saw a significant progress in implantation rate after IMSI in male factor patients (23). In contrast, for the same situation, Oliveira et al. did not observe any noticeable differences between IMSI and ICSI procedures regarding fertilization, embryo quality, and implantation and pregnancy rates, although a trend toward better outcomes in ongoing pregnancy, miscarriage, and live-birth rates (27.0% *vs*. 15.5%, 16.7% *vs*. 16.4%, and 27.0% *vs*. 15.5%, respectively) was reported in the IMSI group (24).

Also, studies showed in patients with oligoasthenoteratozoospermia (OAT), IMSI had significantly better outcomes compared to ICSI, not only in terms of increased pregnancy rates, but also lower miscarriage rates (25, 26). In addition, they showed that patients with motile sperm below 0.1×10^6^/*ml* after the swim-up technique indicated a positive influence of IMSI on fertilization, implantation, and pregnancy rates. So, IMSI can be taken up as the treatment of choice in cases of severe male factor infertility (Table 1) (26). Recently, it was observed that sperm selection with MSOME criteria and IMSI can improve the embryo morphokinetics and clinical outcomes in couples with male factor infertility, especially for OAT and T patients (27).

**Table 1.** The effects of IMSI on ART outcomes with different etiologies

**Authors**	**Etiology of infertility**	**Comments**
**Antinori et al. (2008)(25)**	MF	IMSI is more beneficial than ICSI on all patients with severe OAT, regardless of the number of previous IVF failures
**Khattabi et al. (2013) (22)**	MF	IMSI procedure is a valuable option for patients with severe teratozoospermia
**Balaban et al. (2011)(23)**	MF	Significant improvement in implantation rate afterIMSI
**Oliveira et al. (2011) (24)**	MF	No significant difference between ICSI and IMSI procedures, although trend was better in IMSI
**Knez et al. (2011,2013)(33, 34)**	MF	Significantly higher embryo quality and clinical pregnancy rate in the IMSI group
**Leandri et al. (2013) (35)**	MF	Results of IMSI were similar to the ICSI ones with various degrees of sperm DNA fragmentation, nuclear immaturity and sperm morphology
**Zanetti et al. (2018)(36)**	MF	MSOME is a useful tool for the diagnosis of male infertility. Men who had higher MSOME I+II had better ICSI outcomes
**Kim et al.(2014)(37)**	MF	IMSI increased positive clinical outcomes in patients with OAT
**Goswami et al. (2018) (26)**	MF	IMSI improved embryo development and clinical outcomes and can be taken up as the treatment of choice in cases of severe male factor infertility
**Mangoli et al.(2019)(27)**	MF	Sperm selection with MSOME parameters and IMSI can improve the embryo morphokinetics and clinical outcomes in couples with male factor infertility, especially for OAT and T patients
**Bartoov et al. (2003)(9)**	RIF	No differences in fertilization and top-quality embryo rates in both groups. But, higher pregnancy rate with a lower miscarriage rate were achieved in the IMSI group compared to ICSI
**Hazout et al. (2006) (11)**	RIF	Fertilization and cleavage rates and embryo morphology were similar. But, implantation, pregnancy, and birth rates were improved in IMSI group when compared with ICSI
**Setti et al. (2010)(2014)(12, 21)**	RIF	IMSI not only improves the rate of top-quality embryos, implantation, and pregnancy, but also reduces miscarriage rates as compared with ICSI
**Khattabi et al. (2013) (22)**	RIF	IMSI does not improve pregnancy rate in patients with repeated ICSI failures in the absence of severe male factor
**Delaroche et al. (2013)(10)**	RIF	After two or three IVF/ICSI failures, IMSI seems to give better embryo quality and more blastocysts
**Shalom et al. (2015) (28)**	RIF	Superior implantation, clinical pregnancy, and live birth rates in the IMSI group with a lower miscarriage rate
**Gatimel et al. (2016)(30)**	RIF	IMSI does not improve clinical outcomes in couples with two previous ICSI failures
**Marci et al. (2013) (31)**	Unselected couples	IMSI does not significantly improve IVF outcomes in an unselected randomized infertile population
**Setti et al. (2015)(32)**	Unselected couples	Unselected couples with poor ovarian response do not benefit from sperm selection under high magnification prior to ICSI

IMSI: Intra Cytoplasmic Morphologically Selected Sperm Injection. ICSI: Intra Cytoplasmic Sperm Injection. OAT: Oligoasthenoteratozoospermia. MF: Male Factor RIF: Repeated ICSI failure. T: Teratozoospermia

### IMSI in repeated ICSI failure (RIF) cases:

Bartoov et al., for the first time, evaluated 62 couples with at least two prior ICSI failures undergoing IMSI in the following cycles. The matched control group comprised 50 couples under ICSI treatment with previous experience of the same number of ICSI failures in the same center. There were no differences in fertilization and top-quality embryo rates in both groups. However, higher pregnancy with a lower miscarriage rates were achieved in the IMSI group, in comparison to the controls (66.0% *vs*. 30.0%; p<0.01; 33.0% *vs*. 9.0%; p<0.01, respectively) (9). Later, several studies showed patients with two or more previous ICSI failure benefit more from IMSI not only in terms of increased pregnancy, but also lower miscarriage rates (Table 1) (10–12, 22, 28, 29).

In contrast, Gatimel et al. studied 216 couples with two previous ICSI failures and showed that IMSI did not improve clinical outcomes compared to previous ICSI cycle (30). These contradictory conclusions could be described by two reasons; first, the magnification with which sperm selection was done in ICSI was not suitable to observe some of the sperm anomalies that are not evident at ×200 and should be discovered at magnification of ×400, and the second was the characteristics of the male population under investigation. Therefore, more prospective randomized studies should be performed in order to confirm these findings.

### IMSI in unselected couples:

Marci et al. in their pilot study demonstrated that IMSI does not significantly improve ART outcomes in population with unexplained infertility. Especially there were no significant differences in fertilization, implantation and pregnancy rates between IMSI and ICSI in unselected patients (31). Also, the couples undergoing ICSI with poor ovarian response to controlled ovarian stimulation do not benefit from sperm selection under high magnification prior to ICSI (32). Thus, IMSI is not recommended for normozoospermic patients or couples requesting IVF for the first time (Table 1).

### IMSI in patients with increased sperm DNA damage:

Hazout et al. measured sperm DNA integrity in 72 patients under IMSI and ICSI treatment. They observed improvement of implantation and birth rates, not only in patients with sperm DNA damage, but also in those with normal sperm DNA status (11). Also, others recorded similar results in fertilization and good quality embryos in patients with sperm DNA damage between sibling oocytes splitted into ICSI and IMSI groups (38). However, Cassuto et al. showed no correlation between abnormal head shape spermatozoa with high magnification (Score 0) and DNA fragmentation. But, the rate of chromatin decondensation of their score 0 spermatozoa was two times more than the spermatozoa that scored 4–6. Also, there were no expanded blastocysts following the injection of the spermatozoa with the lowest morphology score and these results confirmed each other (39, 40). On the contrary, some studies showed positive correlation between sperm morphology and sperm DNA quality (13, 41, 42). Hammoud et al. showed in patients with high sperm DNA fragmentation, selection of spermatozoa in high magnification and without vacuole increases the selection of spermatozoa with intact DNA (43). So, since during ICSI, the chromatin structure of microinjected spermatozoa is unknown, the use of noninvasive methods like MSOME criteria and IMSI can be effective.

### IMSI in advanced maternal age and pre-implantation genetic screening:

The quality of the oocytes is age-dependent, which affects the ICSI results. Cassuto et al. showed a difference in the embryo quality produced from oocytes of the women below and above 30 years old after IMSI. They showed that when moderate and bad quality spermatozoa were injected, a lower rate of best and good quality embryos developed in the group of older females in comparison with the younger ones. But, when a high-quality spermatozoon (Class I) was injected, the correlation between age and the quality of the oocyte was negligible. Also, the percentage of high-quality embryos between young and older women was not different because these “top quality spermatozoa” do not need any repair (44). In 2011, Figueira et al. also studied the effect of sperm selection with MSOME on the chromosomal status of embryos from couples with advanced maternal age in PGS cycles. The statistics showed the incidence of sex chromosomal aneuploidy and chaotic embryos were significantly higher in ICSI embryos than in IMSI (23.5% versus 15.0%, 27.5% versus 18.8%, respectively). Moreover, the percentage of cycles without embryo transfer was meaningfully higher in ICSI–PGS cycles (11.8% versus 2.5%). Additionally, the authors reported ‘best looking’ spermatozoa seemed to carry a higher proportion of the X chromosome (45). Setti et al. confirmed that the incidence of XX embryos in IMSI cycles was significantly higher than ICSI (46).

On the other hand, Luna et al. (2015) showed the IMSI procedure significantly improved the embryo quality and the implantation rates without affecting the chromosomal status of the embryos. There was a significant difference between IMSI and ICSI techniques including improved embryo quality, implantation, and pregnancy rates and also reduced miscarriage rates in the IMSI group. But, the rate of aneuploidy was equal compared to ICSI. In IMSI procedure, due to a more accurate selection of spermatozoa, the rate of blastocysts formation with the normal chromosome will be higher than ICSI (47).

### IMSI and paternal age:

In response to the question of whether sperm quality is related to the male age, studies compared 30-year-old men with 50-year-old men and showed a decrease in semen volume of 3–22%, a decrease in sperm motility of 3–37%, and a decrease in normal morphology of 4–18% (48, 49). Silva et al. evaluated semen samples from 975 men under IMSI with different ages, two forms of spermatozoa were considered: normal spermatozoa and the ones with large nuclear vacuoles (LNV). The results showed that the percentage of spermatozoa with LNV was considerably higher in the older group than in the younger groups. Furthermore, regression analysis confirmed a reduction in the normal spermatozoa with increasing age (p<0.05). Also, there was a positive connection between the rate of spermatozoa with LNV and male age p<0.05). These results demonstrated a decrease in semen quality following increased age, and supported the routine usage of MSOME for sperm selection in ICSI for older men (50).

### IMSI in azoospermic patients:

Ai et al. studied the effect of IMSI with testicular spermatozoa on the clinical outcome in the azoospermic patients and compared with ICSI. The pregnancy rates were not different between two groups. But, the abortion rate was significantly lower in the IMSI group compared with the ICSI group (4.5% and 11.8%, respectively) (51). In 2015, Gong et al. demonstrated that IMSI can improve the normal fertilization rates in couples with obstructive azoospermia and teratozoospermia and increase the rate of blastocyst formation in azoospermia (52).

### IMSI in patients with globozoospermia and macrocephalic sperm head syndrome:

There was higher chromatin abnormality, DNA damage and apoptosis in the globozoospermic cases that may reflect one of the main etiologies of ART failure (53, 54). Khalili et al. demonstrated the main role of normal morphology of head in the oocytes activation following injection of round-headed spermatozoa (55, 56). In 2011, Sermondade et al. reported a successful pregnancy and live birth following IMSI in a patient with total globozoospermia (57). Chelli et al. examined the chromosomal status of spermatozoa which was selected by MSOME in the patients with macrocephalic sperm head syndrome. They demonstrated that the larger and more abnormal spermatozoa had the most anomalous (Polyploid) chromosomal content and haploid spermatozoa with MSOME could be selected (58).

### Safety:

One of the most important concerns of the treatment team after the introduction of a new technique is to discuss its safety for the next generation. Cassuto et al. (2014) published the first study assessing the birth defect rates in an IMSI offspring and revealed a protective effect for IMSI compared to ICSI (59). Also, Hershko-Klement et al. concluded that IMSI procedure does not involve an increased malformation rate and may offer a reduced anomaly incidence (60). Recently, Gaspard et al. confirmed the results of two studies and showed the malformation rates observed in the IMSI and ICSI groups were insignificantly different (61).

Since IMSI technique offers innovative standards for sperm evaluation that are unavailable in the classic ICSI, it is hypothesized that the IMSI technique, with a more accurate selection of healthy spermatozoa compared to conventional ICSI, reduces the DNA defects and, consequently, reduces anomalies. Further studies are necessary to reinforce this protective effect and to check whether it is related to a specific subpopulation or specific malformation.

## Conclusion

Selection of a good-quality spermatozoon with normal morphology by using IMSI might be beneficial to embryonic development and to increase implantation and pregnancy rates. According to the majority of studies, it is not recommended to use IMSI routinely in the ART program. The couples with repeated implantation failures, patients with severe male factor infertility, advanced male and maternal ages are the populations who will have higher chances to conceive from IMSI. It is also recommended that diagnostic morphological evaluation of semen samples with high magnification is done before ICSI/IMSI procedure. Besides, according to the current knowledge, no prenatal or postnatal complications in the mothers and off-spring were reported following the IMSI procedure. The effectiveness of IMSI is still controversial mainly due to differences in inclusion criteria, stimulation protocols, seminal and oocyte qualities and many other confounding variables within the ART program. However, there is no doubt that the use of IMSI techniques can be helpful for some infertile couples to have a baby.
